# Supplementary material for: Evaluation of the outcome of a proposed more physiological bypass surgery technique in morbid obesity: Long term 3 years follows up
Source: Ann Med Surg (Lond). 2022 Nov 26;84:104952. doi: 10.1016/j.amsu.2022.104952 (PMC9758339; doi:10.1016/j.amsu.2022.104952)
Supplement: Supplementary data [file mmc1.docx]

| The STROCSS 2021 Guideline | | |
| --- | --- | --- |
| Item no. | **Item description** | **Page** |
| TITLE | | |
| 1 | **Title**  **Evaluation of the outcome of a** **proposed more** **physiological bypass surgery technique in Morbid obesity: long term 3 years follows up** | 1 |
| ABSTRACT | | |
| 2a | **Introduction** –   - Obesity is a chronic condition that affects millions globally; consequently, bariatric surgery is the key to this serious issue. Bariatric procedures are rapidly expanding in number and methods to address the recognized problems. So, it would make a sense for surgeons and patients if there is a more physiological bypass surgery technique in Morbid obesity. This study aims to evaluate the outcome proposed technique |  |
| 2b | **Methods** -   - The present study is a retrospective analysis on 256 participants subjected to the proposed bypass procedure from December 1999 to January 2017, the participants were followed up for an interval of 3years. |  |
| 2c | **Results** - The findings of the present study revealed a significant Excess Weight Loss (EWL). In addition, patients experienced decreased calcium, iron, vitamin B12, Hemoglobin, zinc, and Prothrombin Concentration. However, three to six months after surgery, they experienced a significant improvement until they reached normal levels without any supplement, with a three-year follow-up. |  |
| 2d | **Conclusion**  This proposed Bypass Operation aims to adequate digestions as well as selective absorption without inducing any vital deficit. Most of study's population showed no elements inadequacies, although few percentages emerged during the interval of maximal weight reduction, and it were transient and minimal. No minerals or vitamins were needed.  **Keywords:** Morbid obesity; EWL; physiological bypass surgery; proposed technique. |  |
| INTRODUCTION | | |
| 3 | **Introduction** –   - The resolution of the world epidemic of morbid obesity is debatable, and it is still impossible to find a drug in laboratories. Consequently, bariatric surgeries are the most successful method for EWL as well as managing comorbid metabolic diseases nowadays. - Basically, surgical operations for morbid obesity include complete restriction of food such as gastric band as well as sleeve gastrectomy, in addition to a combination of restriction and reducing intestinal absorption restrictive/mal- malabsorptive RYGBP and BPD-DS. The most effective durable malabsorptive operations carry the nutritional complications that may make patients suffer for life. Because of the feasibility of restrictive gastric operations as the gastric sleeve is done in a wide range worldwide. A lesson learned from redo surgery changed the pendulum to swing away from pure restriction toward malabsorptive procedures 1. - Clearly, medical management as well as follow-up of cases that underwent bariatric surgery present a challenging chance for a qualified gastroenterologist to assess and treat eating disorders, potential nutritional deficiencies, elevated liver enzymes, dysmotility syndrome, as well as psychosocial disorders. - Eventually, Following bariatric surgery, cases usually experience dietary deficiencies and vomiting due to Malabsorption as well as food intolerance 2,3. Up to date there is no bypass surgery operations that could be described as the ideal physiological technique. Bariatric surgeries should not have reached a plateau and surgeons must think and propose ideas that overcome the post operative nutritional problems. - Actually, the idea beyond this technique which proposed by first Author, (El-Banna technique) is to lose weight effectively and safely, along with adequate digestion as well as selective absorption, with no vital post-surgery nutritional deficiencies. |  |
| METHODS | | |
| 4a | **Registration**  *researchregistry8311, and you will find your registration here: https://www.researchregistry.com/browse-the-registry#home/* |  |
| 4b | **Ethical approval**   - The present study was carried out after obtaining the Institutional Board committee's ethical approval at Al- Azhar University Hospitals. We collected an informed written consent form signed by each subject after explaining all the details of the procedure as a new one. All patients signed an Informed Consent Form prior to enrollment in the study. This study was carried out in accordance with the principles of the Declaration of Helsinki (1964) and its later versions. |  |
| 4c | **Protocol**   - This is a retrospective study of cases subjected to the proposed Elbanna bypass procedure operation4 from December 1999 to January 2017. Prior to surgery, we reviewed patients' data in gastroenterology and Bariatric Units of Al-Azhar University Hospitals- faculty of Medicine, Al-Haram Hospital Bariatric Centre, and other private centres in the Arab Republic of Egypt. The present study was carried out after obtaining the Institutional Board committee's ethical approval at Al-Azhar University Hospitals. We collected an informed written consent form signed by each subject after explaining all the details of the procedure as a new one |  |
| 4d | **Patient and public involvement in research**   - Of 279 patients who underwent a new proposed bypass surgery, study recruited 256 patients; 200 females (78.2%) and 56 males (21.8%). All were subjected to the proposed bypass procedure while 23 patients were not subjected to one or more of the follow-up evaluations, and hence they were excluded from our study. |  |
| 5a | **Study design**   - This is a retrospective study of cases subjected to the proposed Elbanna bypass procedure operation4 from December 1999 to January 2017. |  |
| 5b | **Setting and timeframe of research** – comprehensively describe:   - This is a retrospective study of cases subjected to the proposed Elbanna bypass procedure operation4 from December 1999 to January 2017. Prior to surgery, we reviewed patients' data in gastroenterology and Bariatric Units of Al-Azhar University Hospitals- faculty of Medicine, Al-Haram Hospital Bariatric Centre, and other private centres in the Arab Republic of Egypt. The present study was carried out after obtaining the Institutional Board committee's ethical approval at Al-Azhar University Hospitals. We collected an informed written consent form signed by each subject after explaining all the details of the procedure as a new one |  |
| 5c | **Study groups**   - Obese patients’ males and females aged between 18-60 years with BMI above 40 or above 35 with comorbidities. |  |
| 5d | **Subgroup analysis** – comprehensively describe:   - Planned subgroup analyses - Methods used to examine subgroups and their interactions |  |
| 6a | **Participants**   - Inclusion criteria: - Obese patients’ males and females aged between 18-60 years with BMI above 40 or above 35 with comorbidities. - Exclusion criteria: - Age below 18 or above 60, BMI below 35, and unfit for surgery. |  |
| 6b | **Recruitment** –   - Of 279 patients who underwent a new proposed bypass surgery, study recruited 256 patients; 200 females (78.2%) and 56 males (21.8%). All were subjected to the proposed bypass procedure while 23 patients were not subjected to one or more of the follow-up evaluations, and hence they were excluded from our study. |  |
| 6c | **Sample size** – 279 patients who underwent a new proposed bypass surgery, study recruited 256 patients; 200 females (78.2%) and 56 males (21.8%). All were subjected to the proposed bypass procedure while 23 patients were not subjected to one or more of the follow-up evaluations, and hence they were excluded from our study. |  |
| METHODS - INTERVENTION AND CONSIDERATIONS | | |
| 7a | **Pre-intervention considerations** –   - Age below 18 or above 60, BMI below 35, and unfit for surgery. |  |
| 7b | **Intervention** – Transection of the jejunum is done at 50 cm from the dudenojejunal flexure. Re-anastomosis is achieved between the terminal ileum and the proximal jejunum at 100 cm from the ileocaecal valve side to side. Subsequently, the fundus of the stomach was removed, and a band of PTFE is inserted near the 2nd transverse vein traversing to the stomach from the lesser curve making a 30 cm pouch. Band circumference ranged from 7.5 in females to 8.5 in males  So, technique preserved 100 cm of the terminal ilium, proximal 50 cm of the jejunum, and the duodenum. That contribute to the physiological absorption. Also, the anatomical biliary outflow and enterohepatic circulation are significantly augment the procedure benefit and keep the normal digestion. Furthermore, the essential impact of fundal resection on hunger and satiety is adopted during maximal weight reduction. |  |
| 7c | **Intra-intervention considerations** – The idea behind the proposed Elbanna bypass4 operation is to have the following concepts:  1. First Concept: Near normal Digestion and Absorption:  Technique described above does not interrupt the physiological digestion and absorption in the stomach and intestine. Digestion starts chemically with HCL & pepsin, as well as Duodenum, trypsin, chymotrypsin (pancreatic juice), and carboxypeptidase peptidase (in the brush border of epithelium), splitting polypeptides into free amino acids that become ready for absorption. The enterocytes that line the small intestine villi, mostly in the jejunum and the (Duodenum), perform the last step of protein digestion in the intestinal lumen. Free form amino acids chelate minerals in the Duodenum 6,7. Obviously this proposed technique could have the most physiological set for digestion and absorption.  2. Second Concept: Keeping the control of Hunger and Satiation mechanism: Ghrelin, the orexigenic solid, is a key target for satiety and hunger pain control, and nearly 90% are secreted from the fundus which is resected in this technique.  3. Third concept is minimizing food passage by Band: Band used in the technique is to control the proportion of food ingested and slow the passage of food from the pouch to the stomach, even if sometimes, the pouch enlarged after a long time.  Band also helps to prevent GERD, and even if occurred HCL will be in contact with the normal gastric mucosa. |  |
| 7d | **Operator details** –   - Transection of the jejunum is done at 50 cm from the dudenojejunal flexure. Re-anastomosis is achieved between the terminal ileum and the proximal jejunum at 100 cm from the ileocaecal valve side to side. Subsequently, the fundus of the stomach was removed, and a band of PTFE is inserted near the 2nd transverse vein traversing to the stomach from the lesser curve making a 30 cm pouch. Band circumference ranged from 7.5 in females to 8.5 in males - So, technique preserved 100 cm of the terminal ilium, proximal 50 cm of the jejunum, and the duodenum. That contribute to the physiological absorption. Also, the anatomical biliary outflow and enterohepatic circulation are significantly augment the procedure benefit and keep the normal digestion. Furthermore, the essential impact of fundal resection on hunger and satiety is adopted during maximal weight reduction. |  |
| 7e | **Quality control** –   - The revision and coding of data were performed, then they were fed into the computer, as well as via the 26th version of SPSS for Windows (SPSS Inc, Chicago, IL, USA). The normality of quantitative data was tested utilizing the Shapiro-Wilk test and subsequently expressed in the form of standard deviations (SD) and medians. Repeated measures ANOVA test was utilized for the comparison between quantitative variables with post hoc Bonferroni tests. Qualitative data were expressed in the form of percentages (%) and frequencies (n). Chi-square and Fisher exact tests were adopted in order to verify the correlation between qualitative variables. The level of significance was determined at P-value ≤ 0.05. |  |
| 7f | **Post-intervention considerations** –   - Study followed the participants for 3 years following the novel procedure. In addition, we assessed the nutritional elements, eating disorders, liver function, vomiting, as well as other complications following the procedure. EWL, in addition to the assessment of albumin, B12, Hg, zinc, iron, calcium, and PC levels, were all performed during the operation time, 3, 6, 12 months after surgery, as well as then every year for three years. |  |
| 8 | **Outcomes** – Study findings are showed in (Figures: 2-9 and tables 1-5).  For EWL, there were substantial differences between all follow-up times using pairwise comparison with p-value <0.0001.  There was a Significant EWL following surgery after three months (35%), six months (57%), one year (71%), two years (79%), three years (80%), followed by approximately a stationary course to the present, whereas 18% cases reached 100% EWL.  With respect to calcium, pairwise tests revealed significant differences between preoperative levels and three-month, six-month, and one-year levels with p-values of <0.0001, <0.0001, and 0.0004, respectively.  Nevertheless, preoperative levels did not differ substantially between 18-month, two-year, and three-year levels with P-values of 0.081, 0.081, and 1.000, respectively.  On comparing albumin levels by pairwise test, there were significant differences between preoperative levels and three-month and six-month levels with p-value <0.0001.  There were no marked differences between preoperative levels and one-year and 18 months levels with p-values 0.893 and 1.000, respectively. Also, there was a significant improvement in albumin levels from preoperative levels with two years and three years with p-values of 0.016 and <0.0001, respectively.  By setting preoperative iron levels as a baseline, there was a substantial decrease in iron levels in the first three months, six months, and one year with a p-value of <0.0001. In contrast, there was no marked difference in 18-month levels with a p-value of 1.000. Additionally, there was a substantial improvement in the levels of iron at the two-year and three-year follow-up with p-values of 0.006 and <0.0001, respectively.  There was no significant decrease in the first three months in relation to zinc levels with a p-value of 0.436. Nonetheless, there was a considerable decrease in zinc levels at six months with a p-value <0.0001. Also, zinc levels improved substantially from preoperative levels at one year, 18 months, two years, and three five years with a p-value <0.0001.  Regarding HGB levels, there was a significant decrease with a p-value <0.0001 in HGB levels in the first 3 and 6 months, followed by a substantial improvement in HGB levels compared to preoperative levels with a p-value of 0.0004. at 18 months and two-year follow-up, there were no significant differences from preoperative levels with p-value 1.000 and 0.0784, respectively. Finally, there was marked improvement at three-year follow-up with a p-value <0.0001.  VIT B12 levels dropped significantly with a p-value of 0.0001 at three and six months, then improved greatly at subsequent follow-up periods with a p-value <0.0001.  Regarding prothrombin concentration, there were several fluctuations in its levels during follow-up periods: at three months significant decrease with p-value <0.0001, then at six months, no significant difference was found with p-value 0.4306. at one year, there was a considerable decrease again from preoperative levels with p-value <0.0001, in addition to substantial improvement at two years, follow up with p-value <0.0001. at 18 months and three-year follow-up, there were no significant changes with a p-value of 1.000.  Good digestion and selective absorption were the targeted without vital deficiency. Most of the element deficiencies in this study occurred in the period of maximum weight loss due to decreased patients’ tolerability to food caused by small, banded pouch. No vitamins or minerals supplementations were needed; only dietary intake rich in vitamins, proteins, and iron was encouraged.  There is no significant difference between male and female outcomes except the time of menstruation in females, so we advised them to take the dose of vitamins needed by non-obese individuals. |  |
| 9 | **Statistics** – comprehensively describe:   - Statistical tests and statistical package(s)/software used - Confounders and their control, if known - Analysis approach (e.g. intention to treat/per protocol) - Any sub-group analyses - Level of statistical significance |  |
| RESULTS | | |
| 10a | **Participants** – comprehensively describe:   - Flow of participants (recruitment, non-participation, cross-over and withdrawal, with reasons). Use figure to illustrate. - Population demographics (e.g. age, gender, relevant socioeconomic features, prognostic features etc.) - Any significant numerical differences should be highlighted |  |
| 10b | **Participant comparison**   - Include table comparing baseline characteristics of cohort groups - Give differences, with statistical relevance - Describe any group matching, with methods |  |
| 10c | **Intervention** –   - Transection of the jejunum is done at 50 cm from the dudenojejunal flexure. Re-anastomosis is achieved between the terminal ileum and the proximal jejunum at 100 cm from the ileocaecal valve side to side. Subsequently, the fundus of the stomach was removed, and a band of PTFE is inserted near the 2nd transverse vein traversing to the stomach from the lesser curve making a 30 cm pouch. Band circumference ranged from 7.5 in females to 8.5 in males - So, technique preserved 100 cm of the terminal ilium, proximal 50 cm of the jejunum, and the duodenum. That contribute to the physiological absorption. Also, the anatomical biliary outflow and enterohepatic circulation are significantly augment the procedure benefit and keep the normal digestion. Furthermore, the essential impact of fundal resection on hunger and satiety is adopted during maximal weight reduction. |  |
| 11a | **Outcomes** – comprehensively describe:   - Clinician-assessed and patient-reported outcomes for each group - Relevant photographs and imaging are desirable - Any confounding factors and state which ones are adjusted |  |
| 11b | **Tolerance** – comprehensively describe:   - Assessment of tolerability of exposure/intervention - Cross-over with explanation - Loss to follow-up (fraction and percentage), with reasons |  |
| 11c | **Complications** – Complications has been reported as follows: There was no leak at the anastomosis site but occurred from the pouch staples: (2.3%) 6 patients, Bleeding: (3.1%) 8 patients, Infection: (0.8%) 2 patients. Mortality: (1.3 %) 3 patients, Internal Hernia :0 patients (0.0%), Abdominal distension: (31.3%) 80 patients, Incisional Hernia: (1.2%) 3 patients, Vomiting: (41.4 %) 106 patients, Early dumping: (5.1%); 13 patient, late dumping: (2%) 5 patients, Gall stone (0.8 %); 2 patients, Renal stones: (0.8 %); 2 patients, Regain weight: (1.2 %);3 patients. Furthermore, there was no GERD in all the series. |  |
| 12 | **Key results** – comprehensively describe:   - Key results with relevant raw data - Statistical analyses with significance - Include table showing research findings and statistical analyses with significance |  |
| DISCUSSION | | |
| 13 | **Discussion** –   - Basically, the malabsorptive procedure depends on the present concept of maldigestion and thus malabsorption. This concept sacrifices or eliminates stomach portions (85-90%) and bypasses the proximal jejunum as well as duodenum. These components are the most active digestive and absorbent portions of the gastrointestinal system. (Figure:10) - The analysis of serum revealed a continual reduction in albumin in the follow-up interval, which suggests slight depletion of protein in 37 and 38 % of GBP patients and 57 and 52 % of SG cases, respectively 8. - While in current study, there were temporary decreases in albumin levels after three months and six months levels with p-value <0.0001, while no substantial differences were detected between preoperative levels and one year and 18 months levels with p-value 0.893 1.000, respectively. Also, there was a considerable improvement in albumin levels from preoperative levels with two years and three years with p-value 0.016 and <0.0001, respectively. - Really, the loss of fat-soluble vitamins characterizes fat Malabsorption. Patients can present with several problems after the malabsorptive procedure. As a result of malabsorption of fat-soluble vitamins, acute deficiency of vitamin D will occur, which will exacerbate an already compromised capacity to absorb calcium 9. - Within four years following surgery, two-thirds of these individuals will be deficient in fat-soluble vitamins D, A, K, and E. Hypocalcaemia will affect up to half of the patients, and all these patients with low vitamin D levels will develop secondary hyperparathyroidism10. After Gastric Bypass, malnutrition of protein calories may ensue, which can be deadly 11. - Following albumin is a simple way to detect protein deficit. Because the duodenum and jejunum absorb the bulk of protein, malabsorptive bariatric surgical treatments may further lower protein absorption. - Usually, a cohort of cases may experience deficiency of protein following gastric bypass surgery in case the typical limb length is <400 cm. In intractable PD cases following gastric bypass surgery, revision surgery for extension of standard limb length to >400 cm is essential to minimize the complications associated with PD.12 - Ferric iron (Fe+++) in the stomach &duodenal lumen is reduced to its ferrous form by HCL. - Based on a report issued by the American Society of Haematology, individuals subjected to bariatric procedures demonstrate an elevated risk of anaemia, which develops in 33 percent to 49 percent of operated patients within two years following the operation. Gastric capacity and, as a result, hydrochloric acid production and volume are reduced in most bariatric surgery operations 13. - While in this study using preoperative iron levels as a baseline, a substantial decrease in the levels of iron was detected in the first three months, six months, and one year with a p-value <0.0001. In contrast, there was no significant difference with 18 months' levels with a p-value of 1.000. Additionally, there was a substantial improvement in iron levels at two and three years follow up with p-values 0.006 and <0.0001, respectively. - The deficiency of Vitamin B12 is induced by insufficient production of intrinsic factors, low gastric acidity, and most importantly, the bypassing of most of the stomach and duodenum as in combined procedures like RYGBP, approximately one third experience deficiency of vitamin B12 14. - While in this proposed technique, vitamin B12 levels dropped significantly at three months and six months of follow-up with a p-value of 0.0001, then there was significant improvement at further follow-up periods with a p-value <0.0001. - Following bariatric surgery, deficient calcium and vitamin D contribute to rapid bone loss. The prevalence of hypovitaminosis D after surgery ranges from 25% to 73%, based on the follow-up interval 14. - HCl is necessary for the solubilization of calcium, which allows its (active) absorption. Local activation of 25OHD to 1,25(OH)2D3 may be required in human duodenal calcium absorption.15,16 - In this study, pairwise calcium tests showed significant differences between preoperative levels and three months, six months, and 1-year levels with p values <0.0001, <0.0001, and 0.0004, respectively. Nevertheless, preoperative levels did not significantly differ with 18 months, two years, and three -year levels with P-values 0.081, 0.081, and 1.000, respectively. - After RYGB and BPD/DS, multiple prospective case series estimate over 50% of postoperative calcium deficiency.17 That is because of bypassing calcium and vitamin D's primary active absorption sites, namely, the proximal jejunum and duodenum. - The deficiency of zinc is expressed in 42.5perecent of the population at 12 months and afterward was steady. The shortage of zinc was considerably more prevalent following DS, with a frequency of 91.7% at 12 months.18 - Regarding zinc levels, in our study, there was no significant decrease in the first three months with a p-value of 0.436. Still, there was a substantial decrease in zinc levels at six months with a p-value <0.0001. There was a considerable improvement in zinc levels from preoperative levels at one year, 18 months, two years, and three years with a p-value <0.0001 in each one. - Following Roux en-Y gastric bypass, a wide range of research revealed 60-70% vitamin deficiency rates following biliopancreatic diversion, whereas diminished rates of around 10% were reported 19. - Furthermore, Dumping syndrome occurs in up to 40% of patients after Roux-en-Y gastric bypass (RYGB) and in sleeve gastrectomy and up to 50% of patients.20 We have only 13(5.1%) cases of early dumping and 5(2%) cases of late dumping because food passes in a physiological pathway. - GERD: Postoperative bile reflux in the gastric pouch after OAGB is a common finding in scintigraphy and endoscopy. There is evidence of bile reflux roughly in one-third of patients, with one case of oesophageal bile reflux 21. - Reflux symptoms remained or recurred in 55.8% of the participants within the first year after gastric bypass. Within two years after the surgery, 48.8% of participants experienced reflux, with reflux incidence remaining around 50% for up to 10 years after surgery 22. - One year after a successful Roux-en-y gastric bypass operation, Belsey Mark IV fundoplication was performed 23. - In this procedure the band prevents reflux, and if occurred it will be in contact with the gastric mucosa. - Skin Manifestation: Relationship between nutritional deficiency as well as its cutaneous manifestations, biotin, zinc, and protein, for example, cause hair loss. Copper and zinc cause depigmentation and thinning of the skin. Selenium deficiency causes skin cancer, psoriasis, and delayed healing of wounds. Vitamin A or Retinol Xeroderma, Vitamin B2, Vitamin B3, B12, Vitamin B9, and Folic acid 24. - There are no skin affections in our series because there was no vitamins deficiency. - Sometimes, patients subjected to bariatric surgery demonstrate immunological alternations that may finally induce an autoimmune disease 25. No autoimmune diseases were observed in this study. - Even though D-RYGB provided more significant long-term weight reduction, it resulted in maturation of protein calories, which requires periodic revision. Consequently, D-RYGB should not be utilized as the main procedure for superobese or morbid cases.26 Three years in our study EWL (91%) in our study followed by approximately a stationary course until the present, and another 18% of the patients reached 100% EWL. No revision was done. - The determination to avoid or minimize the above-mentioned postoperative complications, Reginaldo supports the modification of the RYGB procedure to maintain the proximal jejunum and duodenum in the food transit. He adheres to the RYGB's technical procedures, and the jejunal loop brought at nearly 25 to 30 cm from the anastomosis with the gastric pouch is latero-laterally anastomosis to the second section of the duodenum. The inclusion of the duodenum utilized as a corrective surgery in cases with acute protein-calorie malnutrition 27. - In this proposed Operation, surgeons preserve nearly most of the stomach, the duodenum, and proximal jejunum, representing the most active parts of the gastrointestinal tract. Therefore, absorption of all macronutrients, micronutrients, and vitamins occurs post-digestion (no pancreatic bile conversion) with premium EWL. There is no toxic colonic or antigenic absorption of the digested protein as well as sterilization by hydrochloric acid, so there is no possibility of autoimmune disease and good liver function while maintaining enterohepatic circulation as 90-95% of bile is absorbed in the terminal iliac. No symptoms or signs of dumping syndrome or stomal ulcer, better satiety, and decreased appetite due to removal of the fundus. No internal hernia because no gastric pouch anastomoses. Single anastomosis. Feasibility of upper gastrointestinal endoscopy. |  |
| 14 | **Strengths and limitations** – More follow up period |  |
| 15 | **Relevance and implications** –   - Consequently, This novel procedure supports the concepts of minimizing ingestion, good digestion, selective absorption and effectively prevents post-bariatric bypass surgery nutritional complications. |  |
| CONCLUSION | | |
| 16 | **Conclusions**   - This proposed Bypass Operation ( Novel Elbanna Bypass Operation)4 aims for selective absorption and good digestion aims to adequate EWL through minimizing food passage, with normal digestions as well as selective absorption without inducing any vital deficit. Consequently, the absorption of all macronutrients, vitamins, and micronutrients occurs perfectly after digestion, along with excellent safe and effective more physiological EWL. |  |
| DECLARATIONS | | |
| 17a | **Conflicts of interest**   - All authors have no conflict of interests. |  |
| 17b | **Funding**   - Self-funding. No funding sources. |  |
| 17c | **Contributorship** Authors: Abduh Elbanna^1^, Ahmed Abd El Aal Sultan^2*^, Yasser Helmy A.,^3^ Ibrahim Bakr^4^, Mohamed Tag El-Din^5^, Mohamed Fathy Labib^6^, Osama Osman Khalil^7^, Mohammed Abd Al-Fattah^8^, Abd Elrahman Safwat El kady^9^, Mohamed I Henish^10^, Hisham W Anwer^11^, Ahmed A. Mostafa^12^, Ibrahim Abdelghaffar^13^, Ahmed Fathy Elhossainy^14^, Mahmoud abd Alhady Abd Al-Aziz^15^, Mohamad Baheeg^16^, Ahmed M. Hegab^17^, Tarek Zaghloul Mohamed^18^, Abdelhamid Hifny^19^, Ayman Helmy Ibrahim^20^, Abd-Elfattah Kalmoush^21^, Marwan Mansour Borham^22^, Alsayed Basiony Moghazy^23^, Mohamed Hassan Elkaseer^24^, Mohamed Sobhy Shaaban^25^ |  |
